# Structural Characterization of Pectic Polysaccharides in the Cell Wall of Stevens Variety Cranberry Using Highly Specific Pectin-Hydrolyzing Enzymes

**DOI:** 10.3390/polym13111842

**Published:** 2021-06-02

**Authors:** Eugenio Spadoni Andreani, Salwa Karboune, Lan Liu

**Affiliations:** Department of Food Science and Agricultural Chemistry, Macdonald Campus, McGill University, S.te-Anne-de-Bellevue, QC H9X 3V9, Canada; eugenio.spadoniandreani@mail.mcgill.ca (E.S.A.); lan.liu@mcgill.ca (L.L.)

**Keywords:** cranberry, pectic polysaccharides, enzymatic fragmentation

## Abstract

The potential of poly- and oligosaccharides as functional ingredients depends on the type and glycosidic linkages of their monosaccharide residues, which determine their techno-functional properties, their digestibility and their fermentability. To isolate the pectic polysaccharides of cranberry, alcohol insoluble solids were first obtained from pomace. A sequential extraction with hot phosphate buffer, chelating agents (CH), diluted (DA) and concentrated sodium hydroxide was then carried out. Pectic polysaccharides present in CH and DA extracts were purified by anion exchange and gel filtration chromatography, then sequentially exposed to commercially available pectin-degrading enzymes (*endo*-polygalacturonase, pectin lyase and *endo*-arabinanase/*endo*-galactanase/both). The composition and linkages of the generated fragments revealed important characteristic features, including the presence of homogalacturonan with varied methyl esterification extent, branched type I arabinogalactan and pectic galactan. The presence of arabinan with galactose branches was suggested upon the analysis of the fragments by LC-MS.

## 1. Introduction

Among plant cell wall polysaccharides, the pectic polysaccharides (PPS) are characterized by high versatility and complexity of their structure, composed of smooth and hairy regions [1,2]. Indeed, the relevant chemical characteristics of PPS include smooth/hairy regions ratio, monosaccharide profile, amounts and distribution of substituents, molecular weight distribution and the typology of side chains they possess. The smooth region consists mainly of homogalacturonan, with smaller amounts of xylogalacturonan; homogalacturonan is a linear polymer of α-(1,4)-d-galacturonic acid [3], which can be partially methyl esterified at the carboxylic group of C6 and acetylated at C2 and C3. While xylogalacturonan has the same backbone structure, with β-xylopyranosyl monomers attached at the C3 of some of the galacturonic acid residues [4]. Rhamnogalacturonan type I (RGI) is the main component of the hairy region, and has a conserved backbone, constituted by the repetition of the diglycosidic unit α-(1,4)-l-rhamnopyranose-α-(1,2)-d-galactopyranosyluronic acid [5]. The galacturonic acid residues are partially acetylated as in homogalacturonan, while the rhamnose residues can be branched at C3 or C4 with oligomers of arabinose and galactose. These neutral branches show great variability between different species, tissues and even in the same tissue in function of physiological changes such as ripening [6,7], but commonly include oligomers of α-(1,5)-l-arabinofuranose (arabinan), of β-(1→4)-d-galactopyranose (galactan), or mixed (arabinogalactans type I and II, which possess a backbone of galactan and arabinose-containing branches). Less common branches have also been reported, such as xylogalacturonan in apple cell wall [8,9], or arabinan with galactose-containing branches in sugar beet, potato and duckweed [10,11], among others. Rhamnogalacturonan type II (RGII) is a much less common but strongly conserved branched region, composed by the repetition of four branches containing neutral and acid glycosidic residues departing from a backbone of homogalacturonan [12].

Smooth and hairy regions are found in variable proportions in extracted plant cell PPS and can be separated from each other only by enzymatic activity or strong chemical treatments. This indicates that they are covalently bound to each other, likely by the galacturonic acid extremities of their backbones [4,5]. While the nature of the bonds between PPS and the other cell wall polymers is still unclear, it has been found that PPS are interconnected with both cellulose microfibrils [13] and xyloglucan [14], forming a complex network in the cell wall. Depending on their chemical structure, PPS can provide functionalities such as gelling, thickening and emulsion stabilizing [5]. As one of the major constituents of soluble dietary fibers, PPS can reduce the risk of cardiovascular disease due to their effects on low density lipoproteins [15]. Furthermore, PPS have been shown to possess in vitro prebiotic properties [16] and have the potential to be utilized for the generation of pectic oligosaccharides with prebiotic [17,18,19] and other health-promoting properties such as antibacterial [20] and antibiofilm effects [21].

Cranberry pomace, obtained during the juice manufacturing process, is a very abundant residual material. This material contains large amounts of cell wall polysaccharides, including PPS. Indeed, cranberry (*Vaccinium macrocarpon*) pomace has been recently studied as a source of antibiofilm oligosaccharides [21,22] derived from xyloglucan and arabinan, but the composition and linkage analyses were limited to the oligosaccharide fractions. The investigation of the structural features of cranberry PPS’s would therefore provide new information, useful to the development of approaches that valorize cranberry pomace as a source of carbohydrate-based functional ingredients. The present study provides a first characterization of some of the PPS obtained from cranberry pomace by chelating agents (CH) and diluted alkaline (DA) extractions [23]. Selected PPS, isolated from the two extracts by anionic exchange and gel filtration chromatography, were analyzed for their monosaccharide composition. Then, these polysaccharides were subjected to the sequential enzymatic degradation with highly specific pectin-degrading enzymatic activities to infer their structural properties from the liberated fragments [24]. The selected pectin-degrading enzymes were homogalacturonan-degrading *endo*-polygalacturonase (EC 3.2.1.15) and pectin lyase (EC 4.2.2.10), followed by a debranching step with *endo*-β-(1,4)-galactanase (EC 3.2.1.89), *endo*-α-(1,5)-arabinanase (EC 3.2.1.99) or the simultaneous application of the two enzymes. The contribution of our findings for the elucidation of the structural properties of RG I in cranberry cell walls was discussed.

## 2. Materials and Methods

### 2.1. Materials

Stevens variety cranberries were provided by Atoka Cranberries Inc., Manseau, QC, Canada and stored at −20 °C. Enzymes were obtained from Megazyme, Bray, Ireland. Analytical grade reagents were from Sigma-Aldrich Co, St. Louis, MO, USA.

### 2.2. Preparation of Alcohol Insoluble Solids

Pomace was obtained from frozen Stevens variety cranberries (Atoka Cranberries Inc., Manseau, QC, Canada) by blending with a Vitamat commercial juicer (Rotor Lips AG, Uetendorf, Switzerland), followed by pressing in cheese cloth for 24 h at room temperature. Pomace was then freeze dried and blended in a Model 7011C commercial blender (Conair, Stamford, CT, USA) with 40 s pulses until it could pass a sieve size of 1.18 mm. Blended pomace was suspended (13.8%, *w*/*v*) in ethanol (95%, *v*/*v*), shaken at 150 rpm for 1 h and filtered on Miracloth rayon-polyester cloth (MilliporeSigma, Burlington, MA, USA). The residues (46%, *w*/*v*) were washed three times with 85% ethanol, 50% chloroform, 50% methanol and acetone, then dried overnight at room temperature yielding the alcohol insoluble solids (AIS).

### 2.3. Sequential Extraction of Polysaccharides

A four-step sequential approach for the extraction of polysaccharides from 8000 mg of dry AIS was implemented adapting the procedure described by Hilz et al. (2005) [25]. Briefly, AIS (2.66% *w*/*v*) were suspended twice in 0.05 M, pH of 5.2 sodium acetate buffer with the addition of 0.05 M ethylenediaminetetraacetic acid (EDTA) and 0.05 M sodium oxalate at 70 °C for 15 min. The suspension was then centrifuged at 8000× *g* for 30 min and filtered on fritted glass funnel of medium pore size, recovering the supernatant (chelating agents extract, CH). The precipitate was washed once with distilled water at room temperature (3% *w*/*v*) to remove excess chelating agents, then suspended twice (2.66% *w*/*v*) in 0.05 M sodium hydroxide at 0 °C for 60 min. The suspension was then brought to pH of 5.2 with acetic acid, centrifuged and filtered, obtaining the diluted alkali extract (DA). The extracts were concentrated by ultrafiltration on a Prep/Scale Spiral Wound module (MilliporeSigma). The retentates were dialyzed with a cut-off of 5000–8000 Da: DA was dialyzed in distilled water for 48 h; CH was dialyzed for 24 h in 0.1 M sodium chloride and 24 h in distilled water. Dialyzed extracts were then freeze dried.

### 2.4. Fractionation of Polysaccharides by Anion Exchange Chromatography

The polysaccharide extracts were fractioned by anion exchange chromatography with an ÄKTApurifier UPC 10, on Source 15Q column (GE Healthcare, Chicago, IL, USA). Elution was conducted at constant flow rate 1.5 mL/min with one column volume of 0.005 M sodium acetate buffer at pH of 5, followed by a linear gradient for 7 column volumes up to 1.5 M. The final molarity was kept for one column volume before re-equilibrating the system with the initial molarity. Fractions containing high concentrations of uronic and/or neutral sugars were pooled, dialyzed in water and freeze dried.

### 2.5. Purification of Polysaccharides by Gel Filtration Chromatography

Selected anion exchange fractions were further purified on Sephacryl S-1000 SF resin (GE Healthcare), a size exclusion chromatography column. The elution was carried out with sodium phosphate buffer (0.05 M, pH 7) containing 0.15 M sodium chloride at a flow rate of 0.5 mL/min. Fractions containing polysaccharides were pooled, dialyzed against distilled water with cut-off 6–8 kDa and freeze dried.

### 2.6. Enzymatic Fragmentation of Selected Polysaccharides

**Figure 1:**
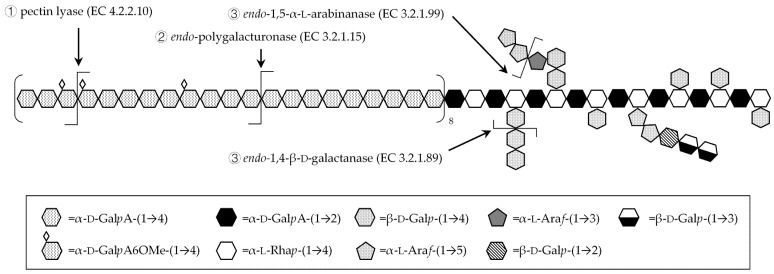
Schematic representation of the structure of the investigated pectic polysaccharides, with examples of the cleavage site of the applied enzymes. ① = enzyme used in the first fragmentation step; ② = enzyme used in the second step; ③ = enzymes used in the third step, separately or in combination.

Purified PPS (0.04 mg/mL) obtained upon gel filtration were suspended in sodium acetate buffer (0.05 M, pH 4.5). Enzymatic reactions were conducted at 40 °C, under 150 rpm shaking in an Excella E24 orbital shaker (New Brunswick Scientific, Edison, NJ, USA). As shown in Figure 1, the enzymatic treatment was first carried with pectin lyase from *Aspergillus niger* (1.4%, *w*/*w* enzyme to polysaccharide ratio). After 42 h of reaction, *endo*-polygalacturonase from Aspergillus aculeatus (1.2%, *w*/*w*) was added to the reaction mixtures, and the reactions were carried out for 72 h. The last stage of fragmentation was conducted with either *endo*-α-(1,5)-arabinanase from *A. niger* (6.25%, *w*/*w*), *endo*-β-(1,4)-galactanase from *A. niger* (0.13%, *w*/*w*) or both enzymes together, for 72 h. Reactions were halted by enzyme inactivation (100 °C for 5 min). Enzymatic reactions were monitored at various times by determining the increase in sugar reducing ends using a 3,5-dinitrosalicylic acid (DNS) test. Briefly, 0.1 mL of aliquots of enzymatic reactions were added to 1.5 mL of DNS 1% *w*/*v* in 1.6% *w*/*v* sodium hydroxide and 0.9 mL of water. After heating at 100 °C for 5 min, 0.5 mL of potassium sodium tartrate 50% *w*/*v* were added. Absorbance at 540 nm was read once the mixture reached room temperature. All reactions were performed in duplicate. Upon the sequential enzymatic fragmentation, the solubilized fragments were separated from polysaccharides by adding an aliquot of reaction mixture (5% *v*/*v*) to ethanol. The mixtures were allowed to settle for 5 h at −20 °C, then centrifuged for 25 min at 6708× *g* to recover the soluble fragments in the supernatant.

### 2.7. Sugar Content and Monosaccharide Profile

Uronic acid content was measured by sulfamate/m-hydroxydiphenyl assay [26]. The phenol–sulfuric acid colorimetric assay was used for the determination of neutral sugar content [27]. In order to determine the monosaccharide profile, fractions were first hydrolyzed using a two-step procedure as previously described [28]. The samples were suspended and incubated at 60 °C for 24 h in HCl/methanol mixture (1:4, *v*/*v*) at a ratio of 0.6% (*w*/*v*) and thereafter boiled for 1 h in trifluoroacetic acid solution at a ratio of 1:8 (*v*/*v*). Hydrolyzed samples were analyzed with high performance anionic exchange chromatography, equipped with a pulsed amperometric detector (HPAEC-PAD) and a CarboPac PA20 column (3 by 150 mm) (Dionex, Sunnyvale, CA, USA). Isocratic elution was performed with 5 mM NaOH (0.5 mL/min). l-Rha, l-Ara, d-Gal, d-Glc, d-Xyl and d-Man were used as standards.

### 2.8. Molecular Weight Distribution Analysis

A high-performance size-exclusion chromatography (HPSEC) system (Model 1525 binary HPLC pump, equipped with a Model 2414 refractive index detector, Waters Co., Milford, MA, USA) was used to estimate the molecular weight distribution in the extracts and their fractions and quantify the yield of polysaccharide extraction. Columns TSK G5000 PWXL and TSK G3000 PWXL (Tosoh Co., Yamaguchi, Japan) were used in series with isocratic flow rate of 0.4 mL/min of 0.1 M sodium chloride. Detector was operated at 30 °C. Dextrans (50–670 kDa) and soybean rhamnogalacturonan (0.3–5 mg/mL) were used as standards for calibration.

### 2.9. Preparation of Methylated PMP-Monosaccharides

Ethanol solutions containing the polysaccharide fragments (0.2 mL) were evaporated at 50 °C until dry in screw cap tubes. The solids were dissolved in 0.5 mL of dimethyl sulfoxide, and 20 mg of freshly powdered sodium hydroxide were added. Iodomethane (0.1 mL) was then added, and the mixtures were gently shaken at room temperature for 10 min. Permethylated samples were recovered by extraction with dichloromethane (1:1. *v*/*v*). The organic phase was washed three times with water and dried. Hydrolysis to monosaccharides was performed by incubation with 0.5 mL trifluoroacetic acid at 100 °C for 1 h. Methylated monosaccharides were then dissolved in 0.05 mL water and derivatized by addition of 0.2 mL ammonia solution (28.0–30.0%) and 0.2 mL of 0.2 M 1-phenyl-3-methyl-5-pyrazolone (PMP) in methanol. The solution was heated at 70 °C for 30 min and dried under nitrogen.

### 2.10. Sugar Linkage Analysis by Liquid Chromatography-Mass Spectrometry

The obtained methylated PMP-monosaccharides were analyzed by liquid chromatography mass spectrometry using an Agilent 1290 Infinity II LC system coupled to the 6545 Q-TOF-MS (Agilent Technologies, Santa Clara, CA, USA). The LC separation was conducted on a Poreshell120 EC-C18 analytical column (Agilent Technologies). The mobile phase A was HPLC water with 5 mM ammonium acetate and the mobile phase B was acetonitrile/methanol mixture (50:50 *v*/*v*) with 5 mM ammonium acetate. HPLC parameters were as follows: injection volume was 1 µL, the flow rate was 0.4 mL/min and the column temperature was set to 35 °C. The mobile phase profile used for the run in positive ion mode was 10% B (0 to 1.0 min), linear increase to 99% B (1.0 to 8.0 min), hold at 99% B (8.0–13.0 min), decrease to 10% B (13.0 to 13.01 min) and, finally, 10% B (13.01 to 16 min). The mass spectrometer was equipped with a Dual AJS ESI ion source operating in positive ionization modes. MS conditions were as follows: for ESI+, the drying gas temperature was 275 °C, drying gas flow rate was 10 L/min, sheath gas temperature was 300 °C, sheath gas flow rate was 12 L/min, the pressure on the nebulizer was 45 psi, the capillary voltage was 3500 V, the fragmentor voltage was 150 V, the skimmer voltage was 50 V and the nozzle voltage was 1000 V. Full scan MS data were recorded between mass-to-charge ratios (*m*/*z*) 100 and 1100 at a scan rate of 2 spectra/s, and were collected at both centroid and profile mode. All ions MS/MS data was collected between *m*/*z* 100 and 1100 at a scan rate of 2 spectra/s for four different collision energies (0, 10, 20 and 40 V). Target MS/MS data were collected using collision energy at 30 V. Reference ions (*m*/*z* at 121.0508 and 922.0098 for ESI+) were used for automatic mass recalibration of each acquired spectrum. Data treatment was conducted using Quantitative Analysis B.07.01 from MassHunter Workstation Software (Agilent Technologies). Three standard oligo- and polysaccharides, raffinose, pectic galactan and pectic arabinan, were used to confirm the retention time and linkage information.

## 3. Results and Discussion

### 3.1. Fractionation and Purification of Cell Wall Pectic Polysaccharides Fractions

The cell wall polysaccharides were isolated from cranberry pomace following a sequential extraction method that involves hot buffer, CH, D, and strong alkali extraction [23]. CH and DA extracts were found to contain abundant uronic acids, indicating the presence of PPS [23]. The fractionation of CH and DA extracts by anionic exchange chromatography confirmed these findings, as it led to the identification of multiple uronic acid-based polysaccharides associated to variable levels of neutral sugars (Table 1). In the CH extract, three main fractions (CH1, CH2 and CH3) were eluted at different ionic strengths. These fractions were characterized by a high content of uronic acid (>90%, mol), indicating the presence of abundant homogalacturonan structures, and taken together they contained 92.5% of the total sugars of the whole CH extract. On the other hand, among the three uronic acid peaks identified in DA extract, the two eluted at lower ionic strength (DA1 and DA2) were accompanied by significantly higher amounts of neutral sugars, while DA3 was comparable with the CH fractions. The fractions rich in neutral sugars represent populations of PPS that possess more neutral branches, such as the arabinan, galactans and arabinogalactans of rhamnogalacturonan type I. Furthermore, the sum of the three pectin-rich DA fractions represented only 67.9% of the total sugars of DA, which also contained neutral polysaccharides. These marked differences in the fractionation of cranberry CH and DA extracts set them apart from those obtained from soybean [29], that presented nearly identical fractionation profile.

The CH extract of grapes showed uronic acid-rich fractions at 0.4 and 0.7 M ionic strength, the latter accompanied by significantly higher proportion of neutral sugars than what was observed in cranberry [30]. Upon anion exchange chromatography, the CH extract of the fruit of *Dacryodes edulis* displayed a small neutral sugar peak at initial ionic strength 0.05 M, and a single major peak containing both uronic and neutral sugars at 0.2 M [31]. DA extracts from *Solanum nigrum* berries displayed two peaks: the major one eluted with distilled water and the second at 0.1 M ionic strength [32].

**Figure 2:**
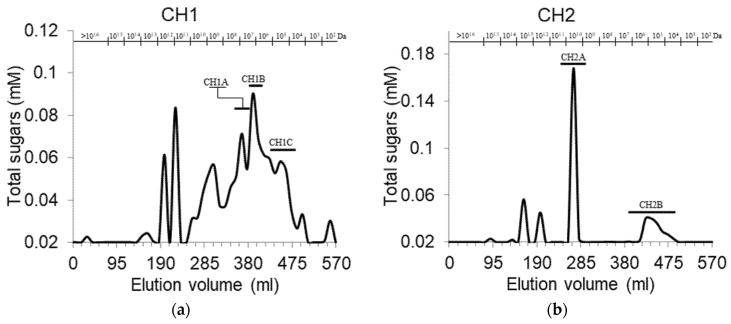

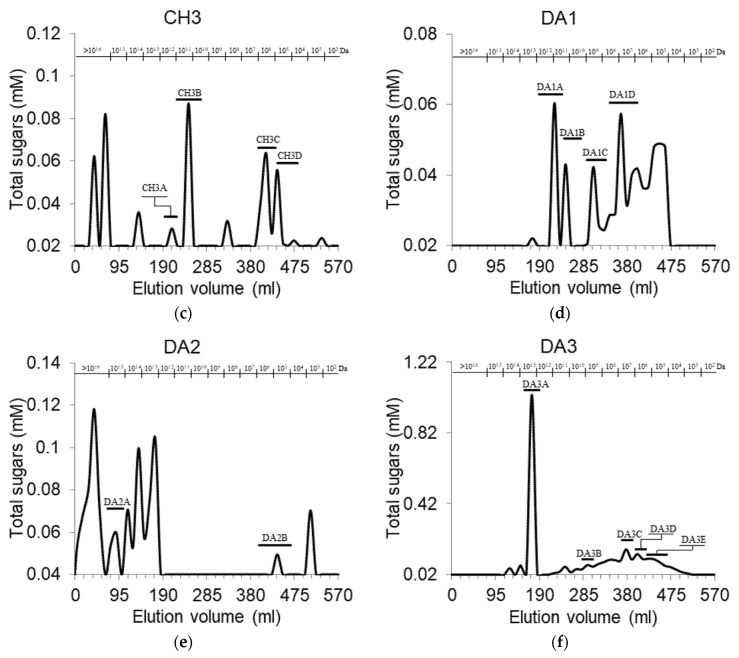
Size exclusion chromatography of selected polysaccharide fractions isolated upon anionic exchange fractionation: (**a**) chelating agents fraction 1 (CH1); (**b**) CH2; (**c**) CH3; (**d**) Diluted alkali fraction 1 (DA1); (**e**) DA2; (**f**) DA3.

The three uronic acid-containing peaks detected in CH and DA anionic chromatography profiles were then pooled and further analyzed by gel filtration chromatography (Figure 2). While all fractions showed polydispersity, the distribution of molecular weights differed. CH1 and DA1 fractions contained high percentages of relatively smaller molecular weight compounds (distributed around 10^7^ Da), while in CH3 and DA2 fractions, higher molecular weights (10^9^ Da and higher) were abundant, and CH2 and DA3 fractions contained one major polysaccharide population, with some much smaller peaks at higher and lower molecular weights. These results suggest that the uronic acid-containing PS eluted at low ionic strength in the anionic exchange chromatography tend to be shorter than those eluted at high ionic strength. The molecular weight of PPS obtained by diluted acids extraction from hawthorn fruit [33] and potato pulp [34], and those found in water extracts from lingonberry [35] were considerably lower (~1.1·10^5^ Da, in the range of 2.0·10^4^ Da to 9.5·10^5^ Da, and ~1.2·10^5^ Da, respectively).

### 3.2. Monosaccharide Profile of Purified Pectic Polysaccharides

Selected uronic acid-rich fractions from the gel filtration were pooled, dialyzed, freeze dried and characterized by monosaccharide profile (Table 2). All fractions contained more than 70.7% of uronic acids, indicating a prevalence of homogalacturonan regions. CH1B, DA2A and DA3A contained the lowest proportions (<50%) of neutral monosaccharides associated to PPS (rhamnose, arabinose and galactose) indicating that in these fractions, neutral pectic structures such as RGI are not as prevalent. Higher proportions of these sugars were detected in CH1C, CH2A and DA3D. The ratio of arabinose and galactose to rhamnose was highest in CH2B (6.42) and DA2A (5.5), suggesting the presence of more or longer branches in these fractions. CH3B, CH3D and DA3D fractions, characterized by the lowest percentages of uronic acids, showed the highest contents of monosaccharides associated with hemicellulose. This suggested the presence of hemicellulose fragments coeluting with these PPS. The existence of covalent bonds between pectic and non-pectic polysaccharide in plant cell wall has been suggested by several studies [14,36,37].

### 3.3. Fragmentation with Homogalacturonan-Degrading Enzymes

**Figure 3:**
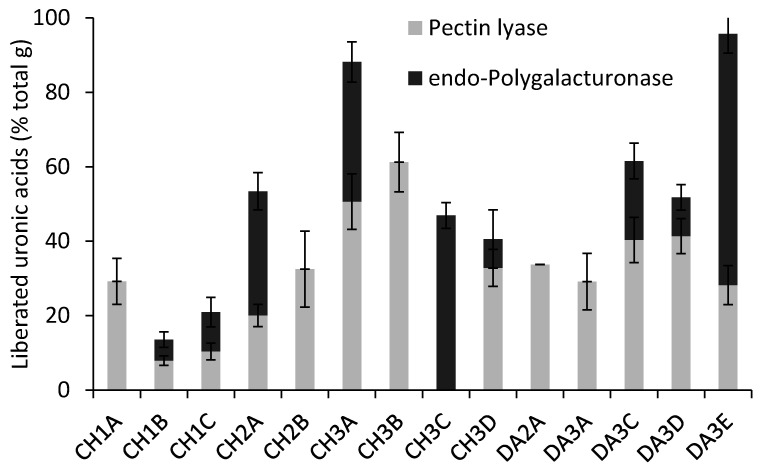
Percentage of the fractions’ total uronic acids liberated by pectin lyase and *endo*-polygalacturonase sequential treatments.

The PPS were subjected to sequential enzymatic treatments with pectin lyase and *endo*-polygalacturonase, which have higher substrate specificities towards homogalacturonan backbone. Indeed, pectin lyase hydrolyses the glycosidic bonds between α-(1,4) bound galacturonic acid methyl ester residues of homogalacturonan, while *endo*-polygalacturonase hydrolyzes the same glycosidic bonds between non-methylated residues. As both pectin lyase and *endo*-polygalacturonase act synergistically to hydrolyze homogalacturonan to alcohol soluble fragments, the release of uronic acid residues (Figure 3) can be correlated to the homogalacturonan proportion in the PPS.

In fractions CH3A and DA3E, that represent PPS eluted at high ionic strength, uronic acids appeared to be almost entirely part of homogalacturonan, as an average of 88.2% and 95.7% of their total amount appeared in the supernatant at the end of the two enzymatic treatments. On the other hand, fractions that were eluted at lower ionic strength, such as CH1A, CH1B, CH1C and DA2A appeared to possess larger amounts of other uronic acid-containing structures (possibly rhamnogalacturonan and xylogalacturonan backbones). In comparison sugar beet pectin, hydrolyzed by pectin lyase, released 78% of its total content of uronic acid [24].

Comparing the amounts of uronic acids released by each enzyme provides information on the abundance of methylated galacturonic acid residues in homogalacturonan. Since all fraction except CH3C released detectable amounts of uronic acids when treated with pectin lyase, it could be concluded that methylation is abundant, with some differences between fractions. As no increase of uronic acid could be observed with *endo*-polygalacturonase treatment of CH1A, CH2B, CH3B, DA2A and DA3A, these fractions appeared to contain high amounts of methylated galacturonic acid in their homogalacturonan, with the non-methylated residues being scattered across the polymer, and thus rendered alcohol-soluble as short oligomers with methylated residues at their extremities. Interestingly, CH3B and DA3A were eluted at high ionic strengths, and as such were expected to contain high amounts of acid residues. The content of non-esterified galacturonic acid that could not be released upon *endo*-polygalacturonase treatment, as it is part of other non-homogalacturonan structures, may account for this discrepancy. On the other hand, in samples such as CH3C, DA3E, CH2A and CH3A, a large portion of the total uronic acid got released by polygalacturonase, indication of the presence of clusters of non-methylated residues in their homogalacturonan chains, since the action of pectin lyase is unable to fragment all the chain into alcohol-soluble oligomers. The proportion of uronic acids liberated by the two enzymes can hence be used as an estimate of the proportion of clusters of non-methylated galacturonic acid residues. As such the fractions DA3E, CH2A and CH1C are expected to be richer in non-methylated homogalacturonan than CH3D, DA3D and DA3C. Onion PPS were found to liberate 62.5% of the total uronic acids, when treated with pectin lyase, while only 18% with *endo*-polygalacturonase [38].

### 3.4. Fragmentation with Debranching Glycosyl-Hydrolase Enzymes

**Figure 4:**
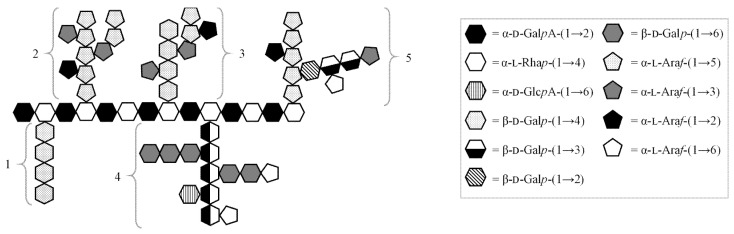
Schematic structure of rhamnogalacturonan type I backbone and major branches [4,5,11]. 1: galactan; 2: arabinan; 3: type I arabinogalactan; 4: type II arabinogalactan; 5: galactoarabinan.

To identify and fragment the RGI neutral sugar branches the fractions, after pectin lyase and *endo*-polygalacturonase treatments, were further subjected to enzymatic treatments with *endo*-α-1,5-arabinanase and *endo*-β-1,4-galactanase either individually or in combination. These two enzymes were selected as a majority of the known most common neutral branches of RGI can be hydrolyzed by their activity (Figure 4) [5].

In fact, *endo*-α-1,5-arabinanase has high specificity towards the hydrolysis of 1,5-bound α-l-arabinofuranose residues, suited for fragmenting the backbone and longer branches of pectic arabinan, as well as the branches of type I arabinogalactan. On the other hand, *endo*-β-1,4-galactanase displays high specificity towards the hydrolysis of 1,4-bound β-d-galactopyranose residues, and as such can hydrolyze the backbones of type I arabinogalactan and pectic galactan. The backbone and branches of type II arabinogalactan, which is occasionally found as sidechain of RGI [39], cannot be hydrolyzed by these enzymes as it is a galactose polymer with β-1,3 and β-1,6 linkage.

The fragments generated by the debranching step were hydrolyzed and analyzed by HPAEC-PAD to determine the amount of released RGI-associated monosaccharides (arabinose, galactose and rhamnose) (Table 3). *Endo*-α-1,5-arabinanase displayed a generally low ability to release arabinose from most of the fractions, as no arabinose was detectable after this enzymatic treatment. The maximum arabinose percentage was released from DA3E (10.8%), followed by DA3D and DA3A. This suggests that, in most fractions, arabinose is not α-1,5-linked into arabinan, but is rather found in other types of neutral branch. In fact, comparison with the amount of arabinose found in the supernatants after *endo*-β-1,4-galactanase treatment shows that the latter was higher for most of the fractions, except CH1C, DA2A and DA3E, with maximum in DA3A (18.0%) and CH3A (15.5%). As more arabinose got liberated upon hydrolysis of the glycosidic bond of β-1,4-galactan, type I arabinogalactan that possesses arabinose decorations [5] may be abundant in the cranberry PPS. Indeed, a type I arabinogalactan structure which is commonly found in the cell wall of other fruits such as orange [40], could be inferred to be present in larger amounts than arabinan. In extracts from rabbiteye blueberry, a fruit of the same genus as cranberry, it was similarly found that the most abundant type of neutral branch is type I arabinogalactan [41]. *Endo*-β-1,4-galactanase hydrolysis was found to be more effective than *endo*-α-1,5-arabinanase, as it released galactose in detectable amounts from all fractions except CH3D, the maximum being in DA3E (8.2%) and CH1B (5.8%).

A portion of galactose may also be included in structures that are resistant to the two enzymes, such as monomeric decorations on RGI backbone as well as type II arabinogalactan. As type II arabinogalactan is often found to possess branches containing 1,6-linked arabinose [3], some of the undetected arabinose may be also part of this structure. The results (Table 3) also show that detectable amounts of galactose were found after treatment with *endo*-α-1,5-arabinanase; structures with arabinan backbone and galactose-containing branches may be present, such as galactoarabinan [6]. While less common in plants than arabinan and arabinogalactan, galactoarabinan have been proposed as RGI branches in sugar beet, where small amounts of galactose could be found in the fragments generated by arabinanase [10], as well as in potato [42], in which 1,4-linked galactan was released by arabinanase, and duckweed [11], in which galactan is 1,3-linked.

The simultaneous treatment with both debranching enzymes resulted in an increased abundance of arabinose and galactose in the fragments of the CH1A, CH2A, CH3D and DA3D. This effect can be used as an indication of the presence of structures, such as type I arabinan, in which both 1,5-linked arabinan and 1,4-linked galactan coexist, and the hydrolysis of the arabinan branches allows better access of galactanase to the backbone. It should be noted that, aside from CH1A, the samples in which this effect was observed were among those with the highest proportion of arabinose and galactose (Table 2). It is therefore possible that in these samples the neutral sidechains are abundant enough to allow detection of the synergistic effect, while for the others the concentration is too low.

### 3.5. Glycoside Linkage Analysis of Polysaccharide Fragments Recovered upon Debranching

The polysaccharide hydrolysates generated upon debranching by glycosyl hydrolase enzymes were analyzed by LC-MS after conversion to methylated and PMP-labeled monosaccharides. Table 4 shows the relative molar proportions of the linkages present in the fragments obtained upon enzymatic debranching of CH1B, DA4A and DA4E.

Although the presence of glycosidic residues involved in multiple glycosidic bonds was likely overestimated, due to the incomplete methylation of the residues, the characteristics of the polysaccharide fragments were discussed. In all investigated fractions, arabinose and galactose represented most of the detected residues, coherently with the expected hydrolytic activity of the debranching enzymes. Arabinose and galactose occupied slightly larger percentages of the monosaccharide profile of the fragments generated by *endo*-α-1,5-arabinanase than by *endo*-β-1,4-galactanase, as the latter contained higher amounts of glucose, rhamnose and, in the case of CH1B and DA3E, galacturonic acid. The presence of more glucose in the fragments liberated by *endo*-β-1,4-galactanase suggests that non-pectic structures may be associated with pectic galactan and type I arabinogalactan and released upon the enzymatic hydrolysis of those chains. The ratios of 1,3,n-Ara moles to total arabinose moles in the fragments obtained by *endo*-α-1,5-arabinanase provide an indication of the extent of arabinan branching, which resulted higher in DA3E (ratio 0.35), followed by CH1B (0.20) and, finally, DA3A (0.05).

Upon treatment with *endo*-β-1,4-galactanase, fractions CH1B and DA3E released a limited amount of galactose, indicating that in these fractions the β-(1,4)-bound galactopyranose of galactan and type I arabinogalactan may not be abundant. On the other hand, since a large portion of these fragments was composed of arabinose, a type I arabinogalactan with short backbone of galactose highly branched with arabinose and arabinans would fit the observations. Branching would in fact hinder the galactanase activity, and the generated fragments would include some of the arabinose-containing branches. Furthermore, simultaneous addition of both debranching enzymes yielded fragments with more abundant arabinose than those from arabinanase and much more abundant galactose than those from galactanase. This effect suggests that the action of arabinanase significantly improves the activity of galactanase, as would be the case in the presence of arabinan branches on type I arabinogalactan. An analogous synergistic effect between these two enzymes was reported in the hydrolysis of potato type I arabinogalactan [43]. The presence of some 1,2,4,n-Gal linkage in these fragments could also reflect the presence of branching points in the arabinogalactan backbone, as they might represent incompletely methylated 1,2,4-Gal residues.

In all the fractions *endo*-α-1,5-arabinanase released a significant amount of galactose residues, with linkages that reflect the presence of linear (1,n-Gal) and branched (1,n,n-Gal) galactose-containing structures. In particular, the proportion of galactose that this enzyme released from CH1B and DA3E was much higher than the one that galactanase did. Overall, these results characterize the neutral branches of CH1B and DA3E as mainly type I arabinogalactan, characterized by β-(1,4) galactan backbones with extended arabinan branches. Coherently with the analysis by HPSEC, α-(1,5) arabinan backbones with galactan branches are likely present in these fractions, but further analysis of the oligosaccharides in the fragments will be needed to verify the presence of this hypothesized galactoarabinan.

On the other hand, in DA3A fragments, a higher release of galactose residues was obtained upon *endo*-β-1,4-galactanase treatment as compared to CH1B and DA3E fragments. This observation, combined with the lack of detectable galactose residues involved in more than two glycosidic bonds (1,n,n-Gal, 1,2,4,6-Gal and 1,2,4,n-Gal), provides a strong indication that DA3A contains 1,4-bound galactan backbones (pectic galactan), which are characterized by low branching [44]. The presence of arabinose in this fraction suggests that some type I arabinogalactan structures may also be present, possibly analogous to the ones reported in sugar beet, where some arabinans are not connected to RGI backbone directly, but through a galactose residue or very short galactan chain [45].

## 4. Conclusions

PPS were identified in the extracts obtained by chelating agents and diluted alkali extraction from Stevens variety cranberries pomace. Selected molecular weight populations of these PPS were collected, purified and further analyzed to determine the monosaccharide composition and their structures. Methyl-esterified homogalacturonan, type I arabinogalactan, and a smaller amount of linear galactan were identified and discussed, and indications of arabinan structures with galactose-containing branches were found. A deeper characterization of the structures of extractable cranberry pectic polysaccharides is expected to contribute to the understanding of cranberry cell wall architecture and to the development of approaches targeted at the isolation of specific structures of polysaccharides and of the oligosaccharides that can be obtained from their hydrolysis.

## Figures and Tables

**Table 1:** Total sugar content and uronic acid percentage of the fractions obtained by anion exchange chromatography of the extracts.

Fraction	Ionic Strength (M)	Total Sugars (% mol/mol in Whole Extract)	Uronic Acid (% mol)
CH1 ^1^	0.31	33.5 ± 2.9	99.0 ± 0.9 ^a^
CH2 ^1^	0.62	31.5 ± 3.0	91.6 ± 5.2 ^a^
CH3 ^1^	0.97	27.5 ± 5.8	96.1 ± 4.3 ^a^
DA1 ^2^	0.52	24.2 ± 3.7	52.9 ± 9.9 ^b^
DA2 ^2^	0.81	28.3 ± 4.4	54.6 ± 11.8 ^b^
DA3 ^2^	1.08	15.4 ± 2.2	85.6 ± 10.9 ^a^

Values represented as average ± standard deviation; ^1^: fractions from chelating agents extract; ^2^: fractions from diluted alkali extract; ^a,b^: means with different letters are significantly different at *p* ≤ 0.05.

**Table 2:** Molecular weight and monosaccharide composition of selected sub-fractions.

Gel Filtration Fraction	Molecular Weight (Da)	Monosaccharide Profile (% mol/mol)			RGI Branching ^c^
		Uronic Acids	Pectic Neutral Sugars ^a^	Hemicellulose Neutral Sugars ^b^	
CH1A	6.54·10^7^	88.1 ± 6.0 ^d,e^	8.0 ± 1.6 ^e,f^	3.8 ± 0.1 ^h^	3.4
CH1B	1.39·10^7^	93.3 ± 4.4 ^d^	3.0 ± 0.5 ^g^	3.7 ± 0.8 ^g,h^	0.6
CH1C	2.92·10^5^	74.1 ± 0.7 ^d,e^	18.6 ± 3.2 ^d^	7.2 ± 0.7 ^f^	2.8
CH2A	>10^9^	75.2 ± 11.8 ^d,e^	18.8 ± 2.4 ^d^	6.0 ± 3.0 ^e–h^	4.7
CH2B	6.32·10^5^	91.3 ± 2.7 ^d,e^	5.2 ± 0.4 ^f^	3.5 ± 1.5 ^g,h^	6.4
CH3A	>10^9^	90.0 ± 3.8 ^d,e^	5.7 ± 1.9 ^f^	4.3 ± 1.9 ^f–h^	3.4
CH3B	>10^9^	76.8 ± 10.7 ^d,e^	13.8 ± 3.5 ^d^	9.4 ± 2.4 ^d–f^	1.2
CH3C	2.97·10^6^	83.6 ± 6.2 ^d,e^	10.1 ± 1.3 ^e^	6.2 ± 2.4 ^f–h^	3.6
CH3D	6.32·10^5^	70.7 ± 11.5 ^e^	16.4 ± 3.5 ^d^	12.9 ± 2.1 ^d^	2.2
DA2A	>10^9^	89.1 ± 8.6 ^d,e^	3.9 ± 1.4 ^f,g^	7.0 ± 1.3 ^f^	5.5
DA3A	>10^9^	86.0 ± 8.6 ^d,e^	5.1 ± 0.5 ^f^	8.8 ± 0.3 ^e^	1.6
DA3C	3.02·10^7^	88.1 ± 8.2 ^d,e^	8.5 ± 2.8 ^e,f^	3.4 ± 0.5 ^h^	1.7
DA3D	6.43·10^6^	71.7 ± 10.2 ^d,e^	23.0 ± 8.1 ^d^	5.3 ± 1.3 ^f,g^	1.4
DA3E	6.32·10^5^	81.2 ± 7.5 ^d,e^	13.3 ± 3.4 ^d,e^	5.5 ± 2.4 ^f–h^	1.7

All values are expressed as mean ± SD; ^a^: calculated as sum of rhamnose, arabinose and galactose. ^b^: calculated as sum of glucose, xylose and mannose. ^c^: calculated as the ratio of arabinose and galactose to rhamnose. ^d–h^: sample means with different superscript letters in the same column are significantly different (*p* ≤ 0.05).

**Table 3:** Monosaccharide of the fragments recovered after the debranching step (%mol in supernatant/total sugar mol in fraction).

	*Endo*-α-1,5-arabinanase			*Endo*-β-1,4-galactanase			*Endo*-α-1,5-arabinanase + *Endo*-β-1,4-galactanase		
	Rhamnose	Arabinose	Galactose	Rhamnose	Arabinose	Galactose	Rhamnose	Arabinose	Galactose
CH1A	n.d.	n.d.	n.d.	4.9 ± 0.5 ^c–e^	4.4 ± 0.5 ^b–d^	3.2 ± 0.6 ^b–e^	16.0 ± 2.3 ^b^	5.6 ± 1.1 ^b,c^	2.9 ± 0.2 ^b,c^
CH1B	10.6 ± 0.5 ^b,c^	n.d.	3.2 ± 0.6 ^b^	1.5 ± 0.1 ^e^	3.6 ± 0.6 ^b–d^	5.8 ± 0.3 ^a,b^	6.9 ± 0.8 ^c–e^	n.d.	n.d.
CH1C	1.9 ± 0.1 ^e^	0.1 ± 0.0 ^c^	0.1 ± 0.0 ^d^	2.8 ± 0.4 ^d,e^	n.d.	0.1 ± 0.0 ^f^	2.1 ± 0.5 ^e^	0.1 ± 0.0 ^c^	0.1 ± 0.0 ^d^
CH2A	5.8 ± 0.5 ^c–e^	n.d.	n.d.	15.6 ± 2.5 ^a,b^	0.6 ± 0.1 ^d^	0.3 ± 0.0 ^f^	5.0 ± 1.4 ^d,e^	0.4 ± 0.1 ^b,c^	0.5 ± 0.1 ^c,d^
CH2B	n.d.	n.d.	n.d.	1.0 ± 0.2 ^e^	2.4 ± 0.2 ^c,d^	1.7 ± 0.3 ^d–f^	2.4 ± 0.3 ^e^	0.1 ± 0.0 ^c^	0.0 ± 0.0 ^d^
CH3A	2.9 ± 0.4 ^d,e^	0.2 ± 0.0 ^c^	0.1 ± 0.0 ^d^	10.0 ± 0.5 ^b,c^	15.5 ± 3.4 ^a^	1.2 ± 0.1 ^d–f^	6.0 ± 0.4 ^c–e^	0.3 ± 0.0 ^b,c^	0.4 ± 0.1 ^d^
CH3B	29.5 ± 3.2 ^a^	0.3 ± 0.1 ^c^	0.9 ± 0.2 ^c,d^	10.7 ± 2.2 ^b,c^	0.7 ± 0.2 ^d^	0.6 ± 0.0 ^e,f^	4.0 ± 0.3 ^d,e^	0.4 ± 0.1 ^b,c^	0.5 ± 0.0 ^d^
CH3C	0.5 ± 0.1 ^e^	0.3 ± 0.0 ^c^	0.1 ± 0.0 ^d^	4.5 ± 0.7 ^c–e^	0.5 ± 0.1 ^d^	0.7 ± 0.0 ^e,f^	5.0 ± 0.3 ^d,e^	0.2 ± 0.1 ^c^	0.3 ± 0.1 ^d^
CH3D	n.d.	n.d.	n.d.	2.1 ± 0.3 ^e^	0.3 ± 0.0 ^d^	n.d.	10.6 ± 2.1 ^b–d^	0.5 ± 0.0 ^b,c^	0.9 ± 0.1 ^c,d^
DA2A	8.1 ± 1.3 ^b–d^	0.2 ± 0.0 ^c^	0.3 ± 0.0 ^d^	n.d.	n.d.	0.2 ± 0.0 ^f^	2.6 ± 0.7 ^e^	0.2 ± 0.0 ^c^	0.1 ± 0.0 ^d^
DA3A	1.0 ± 0.1 ^e^	1.7 ± 0.3 ^b^	0.8 ± 0.2 ^c,d^	8.9 ± 0.6 ^b–d^	18.0 ± 0.5 ^a^	2.8 ± 0.3 ^c–f^	4.1 ± 0.4 ^d,e^	6.0 ± 0.9 ^b^	7.2 ± 1.3 ^a^
DA3C	n.d.	n.d.	n.d.	20.7 ± 1.8 ^a^	3.2 ± 0.4 ^b–d^	4.7 ± 0.1 ^b,c^	12.5 ± 2.3 ^b,c^	2.7 ± 0.4 ^b,c^	2.5 ± 0.4 ^b,c^
DA3D	9.8 ± 1.7 ^b,c^	5.0 ± 0.3 ^b^	2.1 ± 0.2 ^b,c^	4.8 ± 0.3 ^c–e^	7.7 ± 1.1 ^b,c^	3.6 ± 0.1 ^b–d^	14.6 ± 0.8 ^b^	17.8 ± 3.5 ^a^	3.2 ± 0.2 ^b^
DA3E	12.7 ± 1.1 ^b^	10.8 ± 2.3 ^a^	6.4 ± 0.7 ^a^	18.1 ± 1.9 ^a^	8.6 ± 1.7 ^b^	8.2 ± 1.7 ^a^	24.0 ± 1.4 ^a^	4.9 ± 0.3 ^b,c^	0.8 ± 0.2 ^c,d^

All values are expressed as mean ± SD. n.d.: not detected; ^a–f^: sample means with different superscript letters in the same column are significantly different (*p* ≤ 0.05).

**Table 4:** Glycosidic residue linkages (%mol) of the fragments obtained upon enzymatic treatment of polysaccharides. e-Ara: treatment with pectin lyase, *endo*-polygalacturonase and *endo*-arabinanase; e-Gal: treatment with pectin lyase, *endo*-polygalacturonase and *endo*-galactanase. both: treatment with pectin lyase, *endo*-polygalacturonase and *endo*-arabinanase + *endo*-galactanase.

*m/z*	Linkage Pattern	CH1B			DA3A			DA3E		
		e-Ara	e-Gal	Both	e-Ara	e-Gal	Both	e-Ara	e-Gal	Both
523.3	T-Ara	0.8	0.0	1.1	1.0	7.5	2.2	0.8	2.4	0.3
509.2	1,n-Ara	5.6	22.2	4.0	2.9	19.0	8.2	0.0	0.0	2.8
495.2	1,3,n-Ara	6.4	3.6	4.5	3.4	0.0	8.4	17.9	20.9	6.0
481.2	1,2,3,5-Ara	19.5	50.4	55.7	54.0	7.6	26.6	34.6	57.3	55.7
Total Arabinose		32.3	76.2	65.4	61.2	34.1	45.4	53.2	80.6	64.8
567.3	T-Gal	0.9	0.0	0.4	0.8	8.5	1.3	1.6	0.0	0.0
553.3	1,n-Gal	11.0	0.0	9.4	13.9	26.1	9.3	2.3	0.0	10.8
539.3	1,n,n-Gal	19.6	0.0	8.4	8.1	0.0	13.5	6.6	0.0	8.2
525.2	1,3,4,6-Gal	19.0	0.0	6.5	6.3	0.0	7.7	0.0	0.0	7.6
525.2	1,2,4,n-Gal	9.6	9.6	2.7	3.8	0.0	8.7	32.8	5.6	4.4
Total Galactose		60.0	9.6	27.3	33.0	34.7	40.5	43.4	5.6	30.9
523.3	1,2-Rha	0.8	0.0	1.1	1.0	7.5	2.2	0.8	2.4	0.3
509.2	1,n,n-Rha	5.6	8.0	4.3	2.9	19.0	8.2	0.0	0.0	2.8
Total Rhamnose		6.4	8.0	5.5	3.9	26.5	10.4	0.8	2.4	3.1
567.3	T-Glc	0.6	4.8	1.2	0.6	4.7	2.5	2.7	9.7	0.6
495.2	1,3,4-Xyl	0.0	0.0	0.0	0.5	0.0	0.0	0.0	0.0	0.0
567.2	1,n-GalA	0.7	1.4	0.7	0.8	0.0	1.1	0.0	1.7	0.6

## Data Availability

The data presented in this study are available on request from the corresponding author.
